# Guernsey Breeders Journal

**Published:** 1885-04

**Authors:** 


THE GUERNSEY BREEDERS' JOURNAL.—This is the title of a monthly periodical recently established in Westchester, Pa., as the official journal of the Guernsey Breeders' Association. The object of the publishers is to advance the breeding of thoroughbred cattle, and to call attention to the value of the Guernsey breed. The *Journal* is finely illustrated, and will be mailed to subscribers at the low price of $1.00 per annum. We hope to see it obtain a wide circulation.

